# An Assessment of the Provision of Vaping Products by UK Stop Smoking Services Using Data From Freedom of Information Requests

**DOI:** 10.7759/cureus.110160

**Published:** 2026-06-03

**Authors:** Ian M Fearon, Jordan Millar, John Dunne, Marina Murphy

**Affiliations:** 1 Scientific Research, whatIF? Consulting Ltd, Harwell, GBR; 2 Scientific Research, JBP Associates Limited, London, GBR; 3 Public Health, UK Vaping Industry Association, London, GBR; 4 Scientific Affairs, HAYPP Group, Stockholm, SWE

**Keywords:** cigarette smoking, e-cigarettes, flavours, smoking cessation, vaping products

## Abstract

Introduction

Cigarette smoking is the leading cause of preventable death and disease and arises due to prolonged and repeated inhalational exposure to chemical toxicants found in cigarette smoke. Within this context, vaping products, also termed e‑cigarettes or electronic nicotine delivery systems, have been positioned by public health bodies as being substantially less harmful than combustible cigarettes. Furthermore, evidence also suggests that vaping products may be more effective in supporting smoking cessation than traditional support such as nicotine replacement therapy. The UK National Health Service “Swap to Stop” initiative operationalised a harm reduction approach to support smokers’ use of vaping products in switching from cigarette smoking, by providing vaping starter kits via Stop Smoking Services (SSS).

Objectives

The aims of this study were to assess the provision of vaping products and provider-reported aggregate quit rates for vaping products compared with other forms of smoking cessation support, among UK SSS, using data obtained from Freedom of Information requests.

Methods

Questionnaires were sent to 43 local authorities in the UK and, of these, 31 (72.1%) responded. Two authorities (4.7%) withheld permission for the information they provided to be shared or reused for news/reporting. Consequently, responses from 29 (67.4%) local authorities were analysed.

Results

Vaping products are now provided to smokers by the majority (n = 26; 89.7%) of SSS providers, and this is predominantly through the provision of open systems. The majority of providers provide a variety of flavoured vaping products, including fruit, mint/menthol, and tobacco flavours, and among those providing flavour information, 78.9% of service providers (n = 15) reported that ‘fruit’ flavours were most popular or most frequently provided. Quit rates were variable across the different service providers, ranging from 43% to 77% for vaping products and from 35% to 81% for other forms of cessation support. Among those providers who reported quit rates for both vaping products and other forms of support, the average quit rates were 61.5% for vaping products and 56.2% for other forms of support (p = 0.04).

Conclusions

Overall, these findings support that vaping products may provide smoking cessation support to smokers that exceeds that of other forms of support and that flavoured vaping products are highly provided and utilised by UK SSS providers.

## Introduction

Cigarette smoking is the leading cause of preventable death and disease [1-3], contributing to an estimated seven million deaths annually worldwide [4]. Prolonged and repeated inhalation of more than 6,000 chemicals in cigarette smoke [5] is responsible for smoking‑related disease and is linked to the development of lung cancer, heart disease and chronic obstructive pulmonary disease [1-3,6]. Quitting smoking is the most effective means for a smoker to reduce their health risk [2,7,8]. However, despite widespread intentions to stop smoking, fewer than 10% of smokers stop smoking each year [9]. Vaping products, also termed e‑cigarettes or electronic nicotine delivery systems, deliver nicotine to users but expose them to fewer and substantially lower levels of toxicants than cigarettes [10-13]. Vaping products, therefore, may offer an alternative for smokers who have not achieved cessation success with traditional forms of smoking cessation support such as nicotine replacement therapy (NRT) [14,15]. Increasing evidence supports that the effectiveness of vaping products in assisting with smoking cessation exceeds that of traditional smoking cessation support [16-19]. Furthermore, the harm reduction potential of vaping products has received support from several public health organisations, including the UK Royal College of Physicians, Public Health England, Health Canada, and the New Zealand Ministry of Health [11,13,20,21], and many countries have set regulations permissive to vaping products in order to support their smoke-free ambitions.

In the UK, Stop Smoking Services (SSS) are provided by local authorities (councils), in which these authorities either provide the services themselves or outsource the provision through specialist third-party organisations. While national recommendations are made by the UK Department of Health and Social Care (DHSS) and the National Health Service (NHS) regarding how to provide stop smoking services and what support to offer, each of the 317 local authorities or their third‑party service providers can determine for themselves how to provide stop smoking services. In 2023, the DHSS launched the ‘Swap to Stop’ scheme in which one million smokers, equating to approximately 20% of the UK smoker population, were to be encouraged to swap cigarettes for vaping products [22]. Within this scheme, smokers could receive free vaping starter kits alongside behavioural support. While SSS and the National Centre for Smoking Cessation and Training (NCSCT) support the incorporation of vaping products into cessation pathways, there remains variability in adoption and some lack of consensus among public health stakeholders, reflecting ongoing concerns regarding long-term safety, youth uptake, and mixed messaging in clinical practice. The result is that the rollout of this scheme has not occurred nationwide, since the local authorities or their service providers can determine whether or not to participate in the scheme. As of December 2025, it was reported that approximately 97% of local authorities participated in the scheme, with almost universal provision of vaping products [23]. In a recent analysis of data up to December 2024, the introduction of the Swap to Stop scheme led to a 1.5% increase in the proportion of smokers using vaping products in past‑year quit attempts [24], equating to approximately 125,000 additional quit attempts. Other health authorities have also implemented similar initiatives. Health New Zealand, which implemented a ‘Vaping to quit initiative’ in January 2025 in a pilot programme distributing vaping kits alongside ongoing behavioural support and traditional therapies [25]. Early results showed high uptake and engagement, especially among adults who have not succeeded with quitting smoking by using other methods. Of the 5,261 people who quit smoking in the period from January to August 2025, 1,889 people (36%) used a vaping kit provided through this initiative [25].

The aim of the current study was to assess the provision of vaping products by UK SSS using data obtained under the Freedom of Information Act. It provides insight into the scale of provision, as well as the types and flavours of vaping products supplied. It also provides insight into the effectiveness of vaping products in supporting smoking cessation compared with other interventions including NRT and behavioural support.

## Materials and methods

In July 2024, a Freedom of Information (FOI) request was sent to 43 local authorities across the UK concerning their provision of SSS. The recipients were selected to give wide coverage of UK local authorities, with a focus on major cities and population hubs. The FOI request contained a questionnaire (see the Appendix) requesting information concerning: who oversees the provision of their SSS; whether they currently provide vaping products as a stop smoking tool; what types of vaping devices are provided through the service; whether the vaping products provided are flavoured or flavourless; if flavoured vaping products are provided, which flavour categories do they fall under; which flavour categories are most popular/most commonly provided amongst smokers using their SSS; what is the success rate of vaping products in supporting cessation; what is the success rate when vaping products are not provided to support cessation; whether the vaping products obtained through the Swap to Stop scheme were flavoured; and if so, which flavour category do those products fall into. 

Data from questionnaire responses were collated into a database (Excel for Mac, version 16.108.2; Microsoft Corporation, Redmond, WA, US) for qualitative and quantitative syntheses. For categorical data, these are presented as n (%) responses. When assessing which flavour categories were most popular/most commonly provided, among those providers who reported multiple flavours, the first flavour category indicated was taken to indicate the most popular.

To compare the effectiveness of vaping products with other forms of smoking cessation support, quit rates from providers reporting outcomes for both approaches were analysed using a two‑sided paired t‑test with IBM SPSS Statistics for Windows, Version 31 (Released 2025; IBM Corp., Armonk, New York, United States). While the survey did not assess how each SSS determined quit rates, SSS in the UK primarily assess successful quitting by monitoring smoking status four weeks after a set quit date, using both self-reporting and carbon monoxide (CO) validation to confirm abstinence, in accordance with the guidance specified by the UK NCSCT. 

## Results

Questionnaires were sent to 43 local authorities in the UK and, of these, 31 (72.1%) responded. Two authorities (4.7%) withheld permission for the information they provided to be shared or reused for news/reporting, and one authority refused to respond concerning the use of vaping products by their SSS. Consequently, the final dataset includes responses from 28 (65.1%) local authorities. 

Provision of vaping products by SSS

Among the 28 local authorities responding to the survey, 27 (96.4%) reported that they provide vaping products to support smoking cessation. One (3.6%) stated that while they did not provide vaping products at the time of responding to the questionnaire, they planned to implement a vaping product voucher scheme in the future. Among the 27 services providing vaping products, the most commonly supplied device was an ‘open‑system’ (i.e., a pod or tank device refillable with e-liquid), reported by 23 (85.2%) providers. The provision of closed‑system (i.e., an unrefillable closed pod system) vaping products was less commonly reported, by 2 (7.4%) service providers. One (3.7%) service provider reported providing both open- and closed-system vaping products, and one (3.7%) service provider reported providing a vape pen which was indeterminable whether it was an open- or closed-system vape pen. No SSS (0.0%) reported providing disposable vaping products. 

Provision of flavoured vaping products

Among the 27 SSS providing vaping products, all (100.0%) reported offering flavoured vaping products. Details of flavour categories are shown in Table 1. The most commonly offered flavours were fruit (n = 27; 100%), mint/menthol (n = 26; 96.3%) and tobacco (n = 26; 96.3%). One service provider (3.7%) did not provide a mint/menthol‑flavoured vaping product and offered only fruit‑ and tobacco‑flavoured options, and one service provider (3.7%) did not provide a tobacco-flavoured option and offered only fruit and menthol options. Dessert‑flavoured and energy drink/soft drink‑flavoured options were rarely reported, each provided by a single service provider (3.7%; Table 1). 

**Table 1:** Flavour Categories of Vaping Products Provided to Smokers by Stop Smoking Services. Note: Denominator (27) is the number of stop smoking service providers who provide smokers with vaping products. Service providers could select more than one flavour category.

Flavour Category	N	%
Fruit	27	100%
Desserts	1	3.7%
Mint/menthol	26	96.3%
Tobacco	26	96.3%
Energy drink/soft drink	1	3.7%
Other	0	0.0%

Use of flavoured vaping products

When asked which flavours of vaping products were most popular among smokers or most frequently provided to smokers, 8 of 27 (29.6%) SSS providers either could not or did not provide this information, and one (3.7%) only gave a response specific to a segment of individuals seeking smoking cessation support (pregnant smokers). Among the other 19 service providers, ‘fruit’ flavours were most commonly reported (n = 15; 78.9%). 'Tobacco’ flavoured was less commonly reported (n = 3; 15.8%), and no providers reported that ‘mint/menthol’ flavours were most popular/frequently provided. 

SSS providers were also asked whether the vaping products were sourced through the UK Government’s Swap to Stop scheme. Nineteen (73.1%) reported using the scheme, while the remaining (n = 7; 26.9%) did not. All 19 (100%) providers using the scheme reported receiving flavoured products. Within this group, ‘Fruit’ was universally provided (100%), followed by ‘mint/menthol’ (n = 18; 94.7%), and ‘tobacco’ (n = 17; 89.5%). Provision of all three flavour categories was common. One provider reported receiving only ‘fruit’-flavoured products, while another additionally reported access to ‘dessert’ flavours, alongside ‘fruit’, ‘mint/menthol,’ and ‘tobacco’.

Comparison of quit rates between vaping product-supported smoking cessation and other forms of support

**Figure 1:**
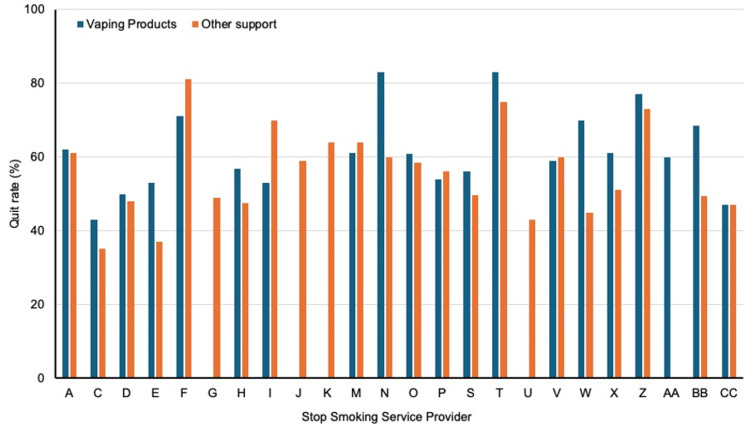
Comparison of Quit Rates Between Vaping Product-Supported Smoking Cessation and Other Forms of Support. Note: data are presented only from those stop smoking service providers who provided information concerning quit rates following the provision of vaping products, other forms of support, or both. The letter codes on the x-axis denote the individual stop smoking service providers who provided quit rate information, which are anonymised.

Information concerning quit rates associated with vaping product-supported smoking cessation and other forms of support (e.g., NRT or behavioural support) is presented in Figure 1. Data were obtained from 20 SSS providers for vaping products and 23 providers for other forms of support (71.4% and 82.1 respectively, of those responding to the survey and allowing their information to be used). Reported quit rates ranged from 43% to 77% for vaping products and from 35% to 81% for other support. Across the 19 providers who provided outcomes information for both interventions, mean (SD) quit rates were 61.5% (11.36) for vaping products and 56.2% (12.57) for other support (p = 0.04; paired t‑test). 

## Discussion

In this study of local authority‑led SSS in the UK, most reported providing vaping products to smokers seeking cessation support. The proportion reporting provision of vaping products (approximately 90%), and the use of the Swap to Stop scheme (approximately 73%), was slightly lower than recent estimates of scheme participation (97%; [23]), likely reflecting ongoing implementation and the continued expansion of service-led adoption during the study period. 

These findings align with broader evidence of increasing integration of vaping products within smoking cessation service provision and their potential contribution to reductions in smoking prevalence in the UK. A recent study reported that the introduction of the Swap to Stop scheme in December 2023 was associated with a 1.5% increase in the proportion of people in England using vaping products in past-year quit attempts, persisting to December 2024 [24]. This increase equates to approximately 125,000 additional quit attempts using vaping products. Alongside individual and population‑level health gains, increased uptake of vaping-supported smoking cessation is also associated with economic benefits. Modelling suggests that a £97 million investment in smoking cessation interventions, above the approximately £280 million currently spent by the UK government, could further accelerate reductions in smoking and generate an estimated £894 million reduction in the burden on public finances by 2030 [26]. By extrapolating this to the £45 million investment by the UK government on the Swap to Stop scheme, the provision of vaping products can be expected to generate a reduction in the financial burden of approximately £414 million within 7 years.

This study also provides evidence that may indicate that the relative effectiveness of vaping products in supporting smoking cessation is greater than that of other forms of cessation support including NRT and behavioural support. Consistent with previous clinical trials and cohort studies, e.g., [16-19], analysis of data from participating local authorities reporting outcomes for both interventions showed that vaping-supported pathways were associated with higher reported aggregate quit rates. Additionally, the study design and means of data collection in this current study provide much‑needed real‑world evidence within routine SSS delivery. While randomised controlled trials (RCTs) remain the gold standard, their generalisability to routine practice can be limited [27]. By collecting evidence outside of the RCT paradigm, we mitigate the issue of generalisability and thus improve our understanding of the real‑world effectiveness of vaping products in smoking cessation. It was clear, however, that some of the local authorities were not capturing quit rates in a manner that could provide data specifically for vaping products and for other forms of support. This is an information gap that must be closed, in order that future studies can better discern the relative effectiveness of different forms of smoking cessation support. Furthermore, the standardisation of data collection frameworks across SSS providers would facilitate better evaluation of the real-world effectiveness of smoking cessation interventions. Such improvements would enhance the ability to identify and recommend best practices to maximise the effectiveness of SSS provision. 

The study also provides insight into the role of flavoured (i.e., non‑tobacco flavoured) vaping products within SSS delivery. While the impact of specific flavours could not be directly assessed, the widespread reporting of preference for or provision of flavoured vaping products (approximately 84% of services) suggests that they are an important component of cessation support. In contrast, tobacco‑flavoured vaping products were only reported as the most popular/frequently provided by approximately 16% of service providers. This finding is consistent with emerging evidence indicating strong user preference for non‑tobacco flavours among former‑smoking vaping product users [28], a preference for sweet/fruit flavours and evidence for switching between flavours during quit attempts [29], an association of the use of fruit‑flavoured vaping products with exclusive vaping product use among former smokers compared with dual‑users [30], and a greater appeal of non‑tobacco flavours which were also found to better promote vaping product uptake and cigarette smoking reduction [31]. However, systematic review evidence concluded that there is currently no clear association between the use of vaping product flavours and smoking cessation or longer‑term vaping product use [29], likely reflecting limited or heterogeneous data. This highlights the need for further research incorporating both RCTs and real‑world evidence such as that presented here, in order to better understand the role that flavoured vaping products may play in smoking cessation. 

Several limitations of this study should be acknowledged. First, response rates were low, meaning that findings may not fully represent the provision of vaping products by UK SSS. Second, the data provide a cross-sectional snapshot of SSS provision in 2024 and may not reflect evolving practice. Given increasing investment and expanding provision of vaping products within SSS, longitudinal research is needed to assess changes over time and to evaluate the effectiveness of vaping product provision in quit attempts and cessation outcomes. Third, in the paper, we compared quit rates for vaping products with 'other support'. There is a heterogeneity in what support other than vaping products may have been provided, both between services and also between different individuals accessing the same SSS. This could be NRT or behavioural support, or a combination of both. Furthermore, multiple forms of NRT could have been used. This is a limitation of this study, and detailed analyses would be required to assess the effectiveness of vaping products in supporting smoking cessation compared with specific other forms of support. Fourth, the survey did not collect data regarding the number of individuals who were provided smoking cessation support by each of the surveyed SSS providers, and in our analyses, we were unable to perform patient-level covariate adjustment. These restrict the interpretation of the statistical comparison of the effectiveness of vaping products compared with other forms of support in smoking cessation. 

## Conclusions

Overall, this study demonstrates that vaping products are widely incorporated into UK SSS, with flavoured vaping products commonly included within cessation support pathways. Among participating providers, vaping-supported pathways were associated with higher reported aggregate quit rates than other forms of support; however, interpretation is limited by the use of provider-level observational data and the absence of patient-level adjustment for potential confounding variables. These findings provide insight into the implementation of the UK ‘Swap to Stop’ scheme and support the need for further longitudinal and patient-level research to better evaluate vaping-supported smoking cessation approaches both in the UK and in other countries, such as New Zealand, which have either implemented similar initiatives or are considering such implementation. 

## Appendices

Supplementary information

Local Authority Stop Smoking Freedom of Information Request Questionnaire 

Dear X,

We are conducting some research on behalf of the UK Vaping Industry Association into the use of vape products by stop smoking services across the country, with a focus on flavours. We are particularly interested in understanding the following about the stop smoking services provided by X.

In your response, please can you also confirm if we have permission to reuse the information provided for news/reporting purposes and as part of communications with the government, civil servants, industry peers and the general public. We will assume that we are able to use the information in these ways unless you explicitly advise us otherwise in your response.

1.    Can you confirm whether the service has any affiliation - in this case meaning receiving funding from, sharing professional resources with, being involved in a partnership with, being controlled/overseen by or having been commissioned by - with any of the following:

The National Health Service (NHS) 

The Department of Health and Social Care 

A local authority council (including county, district, unitary, metropolitan or London borough)

Any other Government department, agency or public group

None of the above

2.    Does the service currently provide vaping products as a stop smoking tool/nicotine alternative to smokers looking to quit with the help of your service?

Yes

No

3.    What type/s of nicotine-containing vape devices are provided through the service?

Disposables

Closed Pod Systems

Open Pod Systems

Other (please specify)

4.    Are the vaping products provided flavoured or flavourless (without flavour additives)?

Flavoured

Flavourless

We do not provide vaping products

5.    If flavoured vaping products are provided, can you confirm which flavour category/categories these fall into:

Fruit

Desserts

Mint/Menthol

Tobacco

Energy Drink/Soft Drink

Other (please specify)

We do not provide vaping products

6.    Without providing specific patient information, please confirm which category of flavour is most popular amongst smokers using the service/which flavour category is most often given out?

Fruit

Desserts

Mint/Menthol

Tobacco

Energy Drink/Soft Drink

Other (please specify)

7.    What is the stop smoking success rate for smokers using the service when vaping products are provided as a quitting tool/nicotine alternative?

8.    What is the stop smoking success rate for smokers using the service when vaping products are NOT provided as a quitting tool/nicotine alternative?

9.    Has the service provided vaping products obtained through the Government’s Swap to Stop scheme?

Yes

No

Don’t know

10. Were the vaping products obtained through the Swap to Stop scheme flavoured?

Yes

No

Don’t know

11. If yes to Q10 above, please clarify which flavour categories these fall into?

Fruit

Desserts

Mint/Menthol

Tobacco

Energy Drink/Soft Drink

Other (please specify)
